# Osteoporosis: a problem still faulty addressed by the Romanian healthcare system. Results of a questionnaire survey of people aged 40 years and over

**DOI:** 10.3389/fmed.2024.1485382

**Published:** 2024-10-23

**Authors:** Narcisa Jianu, Valentina Oana Buda, Denisa Căpățână, Călin Muntean, Teodor Nicolae Onea, Maria Anastasia Jivulescu, Ana Teodor, Mirabela Romanescu, Lucreția Udrescu, Vlad Groza, Mihai Udrescu, Alina Ramona Buzatu, Cristina Adriana Dehelean, Minodora Andor

**Affiliations:** ^1^Faculty of Pharmacy, “Victor Babeş” University of Medicine and Pharmacy, Timișoara, Romania; ^2^Research Center for Pharmaco-Toxicological Evaluation, “Victor Babeș” University of Medicine and Pharmacy, Timișoara, Romania; ^3^Doctoral School, “Victor Babeş” University of Medicine and Pharmacy, Timișoara, Romania; ^4^Faculty of Medicine, “Victor Babeş” University of Medicine and Pharmacy, Timișoara, Romania; ^5^Politehnica University of Timişoara, Timișoara, Romania; ^6^Center for Drug Data Analysis, Cheminformatics, and the Internet of Medical Things, “Victor Babes” University of Medicine and Pharmacy Timișoara, Timișoara, Romania; ^7^Multidisciplinary Heart Research Center, “Victor Babes" University of Medicine and Pharmacy, Timișoara, Romania

**Keywords:** antiresorptive medication, underdiagnosed, knowledge, awareness, prevention, Romanian primary care

## Abstract

**Purpose:**

We aimed to investigate the knowledge and awareness level of osteoporosis, its risk factors, the possible causes of underdiagnosis, as well as the preventive measures and lifestyle behavior of the Romanian population.

**Patients and methods:**

A non-interventional, cross-sectional study was performed, consisting of an in-person survey, in 10 pharmacies located in both urban and rural settings in Romania. The survey was distributed to patients ≥40 years old.

**Results:**

Of 189 respondents, 78.8% were women, the majority age group being 60–69 (31.7%) and 50–59 (30.7%) years old and coming from urban areas (69.3%). Although 75.1% of participants declared knowing about osteoporosis, having a moderate level of knowledge, and women being more aware of the pathology, 77.3% have never performed a DXA test. Moreover, participants already diagnosed with osteoporosis did not show a better disease knowledge than those without a diagnosis. Nearly half of the respondents did not know that a family history of the disease increases the risk of developing it and 60% of them thought that symptoms may develop before a fracture occurs. The preventive strategies tend to be disregarded and thus, underused. Moreover, 42.9% of participants reported being diagnosed with osteoporosis, do not undergo treatment, although they are aware of the existence of effective strategies. The dataset was used to build a participant compatibility network. The network’s clustering revealed six relevant communities, which are not correlated with questionnaire results but reflect the patterns of feature associations.

**Conclusion:**

Preventive and therapeutic osteoporosis education programs are urgently needed in the Romanian population to decrease disability and high mortality risks and thus, to improve the quality of life.

## 1 Introduction

Osteoporosis is a systemic skeletal disease that affects the balance between bone formation and bone resorption, leading to an altered bone density and microarchitecture, and, consequently, increasing the likelihood of fragility fractures. Depending on the underlying causes, osteoporosis is classified as primary or secondary (1–8).

Postmenopausal osteoporosis and age-related or senile osteoporosis are the two types of primary osteoporosis, affecting both women and men (1, 2, 9). Senile osteoporosis may occur in any older adult over the age of 70. It is rooted in some of the aging processes, such as increased parathyroid hormone levels, low-grade inflammatory processes, osteoblast dysfunction, low calcium levels and vitamin D deficiency (2). In the elderly population, bone loss accelerates with increasing age (10, 11). In contrast to postmenopausal osteoporosis, senile osteoporosis associates higher rates of low-bone turnover, increased and, decreased bone formation (12).

Secondary osteoporosis has multiple causes, including endocrine and metabolic disorders, certain diseases, and a number of medicines (1, 2, 4, 9) (i.e., glucocorticoids, proton pump inhibitors, antiepileptics, heparin, lithium, chemotherapy and immunosuppressants, thiazolidinediones, aromatase inhibitors, parenteral nutrition, sodium-glucose cotransporter-2 inhibitors, supraphysiologic doses of thyroid hormones, and selective serotonin reuptake inhibitors used as a long-term treatment or in a high dose regimen) (4, 13).

In recent years, the prevalence of osteoporosis has increased, thereby escalating the economic burden of the disease (4, 14). 18.3% of the world’s population is affected by osteoporosis, women having a higher risk of developing the condition compared to men (9). In Romania, the prevalence of osteoporosis is lower than the European average, with 4.8% of Romanian patients aged 50 years or older being diagnosed with the disease, compared with 5.6%, EU’s average (15). Osteoporotic fractures often lead to pain and disability, and more than 50% of the patients with a hip fracture are unable to regain independently living (2, 4, 13, 15).

One of the most important tools in managing osteoporosis is the assessment bone mineral density (BMD), typically performed using dual-energy X-ray absorptiometry (DXA) scanning. The assessment identifies the patient’s risk level, detects the presence of osteopenia or osteoporosis, guides clinicians to select appropriate medication, and aids in monitoring the disease and effectiveness of treatment. It is estimated that at least 11 DXA machines per million people are required for adequate assessment of osteoporosis and for monitoring patients undergoing treatment. Yet, due to insufficient equipment, Romania falls into the category of European countries lacking proper DXA machines, which may be a cause of the underdiagnosis of osteoporosis (15).

A wide range of drugs have been approved and are available for the prevention and treatment of osteoporosis: bisphosphonates, RANK—ligand inhibitor, selective estrogen receptor modulators, parathyroid hormone analogs, and sclerostin inhibitor (13). However, in Romania, a significant percentage of individuals at high risk of fractures do not receive treatment, the treatment gap among osteoporotic women being 78% in 2019 (15). Despite the existence of clinical guidelines, some patients remain undiagnosed even after experiencing a fracture (13, 15, 16). Moreover, in a previously published STOPP/START v.2 criteria-based study, our research group reported a lack of prescription of antiresorptive or anabolic bone therapy for documented osteoporosis in patients from rural and urban areas of Romania (17, 18).

The aging population (expected to rise to 29% by 2050, compared to 19.7% in 2018 in EU) is the main catalyst for the onset of frailty syndrome, characterised by increased vulnerability due to physical, mental, and social decline (19). Elderly people with poor diet, sedentary lifestyle, and comorbidities such as cardiovascular diseases, osteoporosis, dementia, diabetes mellitus, and cancer are at high risks of developing frailty (20, 21). Features of frailty include limited mobility, susceptibility to falls and fractures, frequent and prolonged hospitalizations, and increased mortality rates (22).

In Romania, CVDs are the leading cause of death, accounting for 59.3% of all deaths nationwide (23). Romania also ranks second in Europe in terms of the proportion of elderly individuals with disabilities, with a large number of people reporting walking difficulties. Hence, effective approaches in the prevention and treatment of osteoporosis in a high cardiovascular risk population will decrease the risk of frailty among elderly Romanians.

Given the aforementioned challenges, we aimed to investigate the level of knowledge and awareness of osteoporosis and associated risk factors among the Romanian population. Our objective was to identify the possible causes of underdiagnosis of osteoporosis via an in-person survey. In addition, since osteoporosis is preventable, we also sought to observe preventive measures and lifestyle behaviors of the study participants. To our knowledge, this is the first Romanian study that intends to evaluate the population’s understanding of osteoporosis and its risk factors. Our work can serve as a starting point for further research and an alarm signal for the general public to better comprehend and manage this pathology, in order to decrease frailty.

## 2 Materials and methods

### 2.1 Study design

A non-interventional, cross-sectional study was conducted over 3 months (from February 1, 2023, to April 30, 2023) in 10 pharmacies located in both urban and rural areas across four Romanian counties: Timiș, Arad, Caraș-Severin, and Olt. The study was designed around a self-administered questionnaire, distributed to patients who visited the community pharmacies included in the research. A total of 189 participants were selected for the study. Written informed consent was obtained from all the respondents.

#### 2.1.1 Inclusion and exclusion criteria

The survey included participants of 40 years old or above, able to read and write in Romanian and willing to fill in the questionnaire. Respondents below 40 years old, those with language barriers or with signs of cognitive impairment, and those unwilling to participate in the study were not included. We also excluded any questionnaires that were incompletely answered.

#### 2.1.2 Sampling methodology of the pharmacies included in the study

First, a representative sample of pharmacies from different areas was established to reflect a balanced urban-rural distribution and the socio-economic diversity of patients. The targeted pharmacies were selected based on predetermined criteria [urban areas: both from in the municipality cities, centrally located, with a high flow of clients/patients from all over the county, recognized as well-stocked, and from smaller towns located at least 50 km from the municipality; rural areas: pharmacies in villages closer to a larger town (about 20 km) and at a greater distance from the town (minimum 30 km)], without subjective intervention by the researchers. Next, the selection of pharmacies was randomized from a complete list of available pharmaceutical establishments in the study region using a randomization algorithm. This process ensured that no pharmacy was included or excluded based on its specific characteristics.

A total of 26 pharmacies were contacted. Ten pharmacies agreed to collaborate and were included in the study, each pharmacy receiving 60 printed questionnaires (600 distributed questionnaires). At the end of the study period, although 375 questionnaires were returned, only 189 questionnaires were fully completed and were included in the study.

### 2.2 Data collection and research tool

A number of pharmacies were invited and agreed to collaborate in the research. Each pharmacy delegated two pharmacists to handle data collection, with the responsibility of informing the participants about the study specifics, providing the questionnaire, and ensuring any participant questions were clarified. After ensuring the participants of complete confidentiality and anonymity of their responses, the written informed consent was obtained. Two designated persons entered the data into a Microsoft Excel Sheet, with each questionnaire assigned a unique identification number to allow for error checking for each respondent. A third person then randomly reviewed the input data to confirm its accuracy. Lastly, the body mass index (BMI—kg/m^2^) was calculated based on the height and weight provided by the participants. Thus, a BMI value <18.5 kg/m^2^ is characteristic of underweight people, a BMI between 18.5 and 24.9 kg/m^2^ is considered as normal weight, overweight is represented by a BMI 25–29.9 kg/m^2^ and obesity is considered as >30 kg/m^2^ (24, 25). The questionnaire was developed based on the European, Canadian and French sources available in the specialty literature (26–30). The final version of questionnaire consisted of 31 items, including both dichotomous and multiple answers questions, and was subsequently pre-tested and validated. The survey began by describing the study’s objective and assuring participants of the confidentiality and anonymity of their responses.

The first part of the questionnaire (questions 1–7) intended to collect socio-demographical data, including age, sex, height, weight, education level, residency, and the residency of their general practitioner (GP). The following section (questions 8–9) evaluated the respondents’ sources of information about osteoporosis (e.g., physician, social networks, family, friends), if applicable.

Next part of the questionnaire (question number 10) concerned 13 items that set out to assess the level of knowledge regarding osteoporosis and its risk factors using “yes or no” responses. Each correct answer was assigned 1 point, while incorrect answers received 0 points, resulting in a maximum score of 13 points. Participants’ knowledge scores were then categorized into three different levels: those scoring 50% or less (fewer than 7 points) were categorized as having low knowledge, those scoring between 50 and 75% (7–10 points) were categorized as having moderate knowledge, and those scoring over 75% (11 points and more) were categorized as having high knowledge.

The middle part of the survey (questions 11–26) collected information on personal medical history, heredo-collateral history, and use of certain medications for more than 3 months. Additionally, it included questions about nutrition and lifestyle, such as calcium and vitamin D intake, daily physical exercise, smoking, alcohol intake, and caffeine consumption habits.

Lastly, the questionnaire (questions 27–31) identified the participants who are at risk of have developed osteoporosis. For those already diagnosed, we gathered data on the administered treatment and we evaluated to which extent the DXA test was performed as a preventive or diagnostic measure.

### 2.3 Validation and reliability

The validity of the survey was determined by a three-stage assessment process with a committee of specialists.

#### 2.3.1 Pre-validation stage

The first version of the questionnaire was drafted by a group of professionals: a general practitioner, a clinical pharmacist, a clinical pharmacy resident and a pharmacy student. A second group of specialists (an endocrinologist, an orthopedist and a public health physician) ensured that the questionnaire is clinically appropriate.

#### 2.3.2 Constructive validation phase

The questionnaire was distributed to a small group of participants (*n* = 10) to solicit input on the questions’ clarity, understanding, and relevance. The suggestions were documented and used to revise and improve the questionnaire.

#### 2.3.3 Empirical validation phase

To assess its psychometric properties, the questionnaires were distributed in 2 rural pharmacies and 2 urban pharmacies (8 questionnaires per pharmacy). Internal consistency and test-retest reliability were determined. Test-retest reliability was calculated through intraclass coefficient (ICC), and the results showed an ICC value of 0.65 and an alpha coefficient of 0.6. This reflects moderate internal consistency and indicates the items are largely homogeneous, supporting the validity of the present questionnaire.

#### 2.3.4 Ethical considerations

This study was conducted in accordance with the Declaration of Helsinki and its latest amendments. Furthermore, it was approved by the Ethics Committee of the “Victor Babes” University of Medicine and Pharmacy (no.47/2024). As aforementioned, written informed consent was also obtained from all respondents.

### 2.4 Statistical analysis

The statistical analysis was performed using Statistical Package for Social Science Version 22 (IBM, Armonk, NY, United States) at a statistical significance level of 0.05 (*p* < 0.05). Kolmogorov–Smirnov test was used to assess data normality. All categorical variables were expressed as number and percentage, whereas quantitative data were represented as mean and standard deviation. The Student *t*-test, ANOVA test, chi-square test and Spearman’s correlation test were used to compare and identify the associations between the knowledge score and the studied variables.

### 2.5 Complex network-based analysis

The network analysis of the osteoporosis dataset assumes the building of a graph $G=\left(V,E\right)$ , where the vertices $v_{i}$ (from the vertex set *V*, $v_{i}\in V$ ) represent the participating individuals, and the undirected edges $e_{ij}$ between vertices $v_{i}$ and $v_{j}$ represent a compatibility relationship between individuals i and j . We then apply network clustering on G , using the energy layout in Mathematica 11.1.1 to generate participant communities (or clusters).

**Figure 1:**
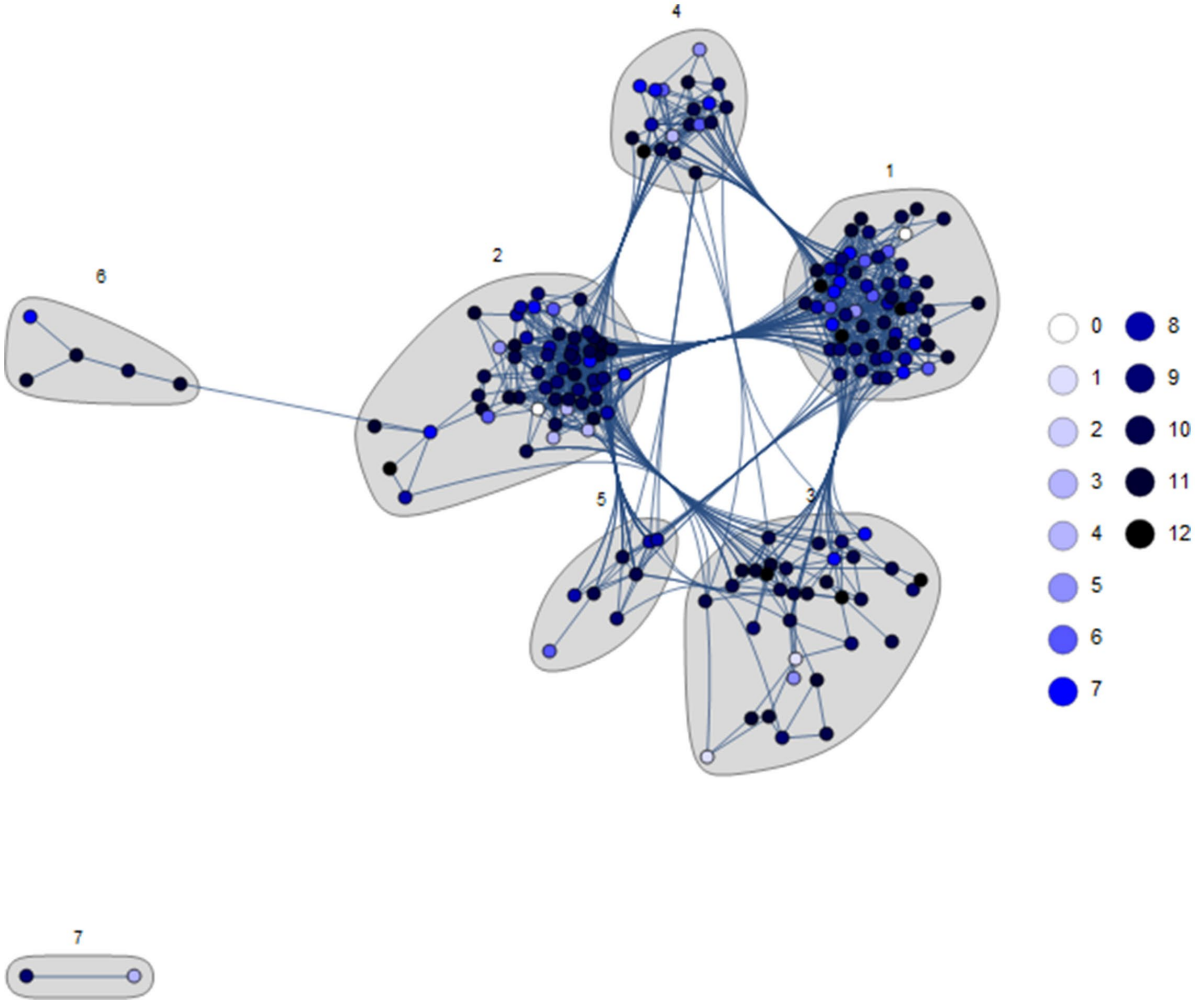
The participants’ network G was built according to participant compatibility relationships and clustered with the energy layout; the node colors represent the participants’ questionnaire results. The number of participants in the dataset is 189; however, 4 vertices have no edge, so the number of vertices in G is |*V*| = 185. The clustering reveals 7 distinct participant communities, emphasized with a gray background. The first 6 communities pertain to the main connected component, while Community 7 is disconnected.

Figure 1 presents the clustered network G, where node colors depict the corresponding participant’s level of knowledge according to the questionnaire results. We define compatibility between two participants based on the individual features recorded in the osteoporosis dataset. These features are classified into 4 classes: anthropometric, demographic, lifestyle, and clinic. Accordingly, two participants i and j are compatible (i.e., we have an edge $e_{ij}$ between $v_{i}$ and $v_{j}$ ) if they are compatible according to at least 3 out of 4 compatibility classes. The anthropometric class comprises the following features: age, sex, and body mass index (BMI). The age is an integer number, but we discretize it by defining 5 age intervals: 40–49, 50–59, 60–69, 70–79, and 80–89. We use 4 discrete BMI levels: <18.5 (underweight), 18.5–24.9 (normal weight), 25.0–29.9 (overweight), and ≥30 (obesity). The demographic class features are the education level (low and high) and living environment (rural and urban). The following characteristics are binary in our analysis: calcium supplements, vitamin D supplements, alcohol, coffee, smoking, physical activity, fracture history, comorbidities, osteoporosis diagnosis, DXA scan, and osteoporosis treatment. We assign the participant compatibility according to the feature class as follows: for the anthropometric class, 2 out of 3 identical features; for the demographic class, 2 out of 2; for the lifestyle class, 5 out of 6; and for the clinic class, 4 out of 5. Our analysis of the cluster structure in Figure 1 excludes the 4 vertices (i.e., participants) without edges, as well as 2 vertices in community/cluster 7 (disconnected from the main connected component). Therefore, our investigation includes the 183 vertices in communities 1–6 from Figure 1. The edge density distribution in network G determines the segregation of communities, while the feature compatibility defines the presence of edges. Consequently, the association of features is linked to the community characterization. Indeed, although many possible combinations exist for the 16 features considered, the main component reveals only 6 communities that exhibit specific feature associations.

## 3 Results

Of the 600 questionnaires initially distributed, 375 were returned, but only 189 were fully completed, and thus, they were included in the present study.

The socio-demographic data of our survey group are systematically presented in Table 1. Women were overrepresented, accounting for 78.8% (*n* = 149) of the 189 respondents, while men made up 21.2% (*n* = 40). The majority of respondents were distributed across the age groups of 50–59 (30.7%, *n* = 58) and 60–69 (31.7%, *n* = 60), indicating a higher representation of middle-aged and senior individuals, while other age groups were less well represented, with 21.2% participants (*n* = 40) of 40–49 group, 14.0% participants (*n* = 27) of 70–79 group and 2.1% participants (*n* = 4) of 80–89 group. The overall mean age was 58.9 years with a standard deviation of 10.24 and the median age was 59 years old, with a range between 40 and 85 years. A larger proportion of participants came from urban areas (69.3%), while the remainder lived in rural areas (30.7%). In terms of education level, the largest share is represented by respondents with high school education (40.2%), followed by those with higher education (34.9%).

**Table 1:** The absolute (count) and relative (%) frequency of socio-demographic characteristics.

Variable	Value	Count (*n*=)	%
Gender	Male	40	21.2%
	Female	149	78.8%
Age range	40–49	40	21.2%
	50–59	58	30.7%
	60–69	60	31.7%
	70–79	27	14.3%
	80–89	4	2.1%
Residency	Rural	58	30.7%
	Urban	131	69.3%
Level of education	Secondary school	25	13.2%
	High-school	76	40.2%
	Post-secondary school	22	11.6%
	University	66	34.9%

Table 2 presents the frequency of responses regarding osteoporosis signs, symptoms, risk factors, family history, prevention, diagnosis, management, and treatment. Although most participants (75.1%) reported having knowledge about osteoporosis, a little more than one-quarter received information about the disease from a physician (26.2%). Instead, social media was the main source of information (27.7%). Moreover, less than a half (39.2%) of respondents had been informed by their general practitioner about the risk of developing osteoporosis.

**Table 2:** The absolute (count) and relative (%) frequency of responses corresponding to osteoporosis signs, symptoms, risk factors, family history, prevention, diagnosis, management and treatment.

Variable	Value	Count (*n*=)	%
Knowledge about osteoporosis	No	47	24.9%
	Yes	142	75.1%
Source of information	Physician	50	26.2%
	Social networks	52	27.7%
	Family/friends	48	25.5%
	Others (radio/TV)	39	20.6%
BMI	Underweight (<18.5 kg/m^2^)	3	1.6%
	Normal weight (18.5–24.9 kg/m^2^)	51	27.0%
	Overweight (25–29.9 kg/m^2^)	84	44.5%
	Obesity (>30 kg/m^2^)	51	27.0%
Recent falls (in the last year)	No	143	75.7%
	Yes	46	24.3%
Bone fractures in the past	No	147	77.8%
	Yes	42	22.2%
Family history of fracture	No	157	83.1%
	Yes	32	16.9%
Taking calcium supplements	No	135	71.4%
	Yes	54	28.6%
Taking vitamin D supplements	No	116	61.4%
	Yes	73	38.6%
Alcohol consumption	No	129	68.3%
	Occasionally	9	4.8%
	Yes	51	27.0%
Caffeine consumption	No	44	23.3%
	Yes	145	76.7%
Active smoker	No	148	78.3%
	Yes	41	21.7%
Daily physical exercises	<30 min/day	86	45.5%
	30–60 min/day	58	30.7%
	>60 min/day	45	23.8%
Family history of osteoporosis	No	157	83.1%
	Yes	32	16.9%
Information on the risk of developing osteoporosis from general practitioner	No	115	60.8%
	Yes	74	39.2%
Osteoporosis diagnosis	No	161	85.2%
	Yes	28	14.8%
DXA bone quality test	No	146	77.3%
	Yes	43	22.8%
Osteoporosis treatment	No	12	42.9%
	Yes	16	57.1%
Type of treatment	Bisphosphonates	10	62.5%
	Denosumab	2	12.5%
	Teriparatide	4	25.0%

Based on the 13 questions in Table 3 and the knowledge cut-off points presented in the methods section, we assessed the participants’ level of knowledge on osteoporosis. Out of the total number of 189 subjects, 126 subjects (66.7%) were identified as having moderate knowledge, 40 (21.2%) as having high knowledge, and 23 (12.2%) as having low knowledge. The overall mean and standard deviation of total knowledge was 9.02 ± 0.15. Individual assessment of responses helped identify the less known information about this pathology among the studied population. Most participants were aware that osteoporosis benefits from effective treatments (98.4%), increases the risk of fractures (94.2%), and is more common in females (91.5%). Conversely, 46.0% of respondents were unaware that a family history of osteoporosis predisposes to the disease. More than half of participants incorrectly believed that osteoporosis causes symptoms before fractures occur (59.3%) and that a fall is not a factor as important as low bone density in the development of fractures (64.0%). Moreover, 72.5% of the responses to the question whether any type of physical activity is suitable for osteoporosis were erroneous.

**Table 3:** Evaluation of individual responses to the osteoporosis knowledge level assessment.

Statement	Statement validity	Number (%) of correct answers	Number (%) of incorrect answers
1. A family history of osteoporosis strongly predisposes a person to this disease	True	*n* = 102 (54.0%)	*n* = 87 (46.0%)
2. Osteoporosis is more common in women than in men	True	*n* = 173 (91.5%)	*n* = 16 (8.5%)
3. There is a small loss of bone mass in the first 10 years after the onset of menopause	False	*n* = 159 (84.1%)	*n* = 30 (15.9%)
4. Osteoporosis increases the risk of bone fractures	True	*n* = 178 (94.2%)	*n* = 11 (5.8%)
5. Osteoporosis causes symptoms (e.g., pain) before possible fractures occur	False	*n* = 77 (40.7%)	*n* = 112 (59.3%)
6. A fall is a factor as important as the presence of low bone density in the occurrence of fractures	True	*n* = 68 (36.0%)	*n* = 121 (64.0%)
7. Starting at age 50, most women can expect at least one fracture over the next few years	True	*n* = 146 (77.3%)	*n* = 43 (22.8%)
8. Smoking can contribute to osteoporosis	True	*n* = 134 (70.9%)	*n* = 55 (29.1%)
9. High salt intake is a risk factor for osteoporosis	True	*n* = 112 (59.3%)	*n* = 67 (40.7%)
10. Any type of physical activity is beneficial for osteoporosis	False	*n* = 52 (27.5%)	*n* = 137 (72.5%)
11. An adequate intake of calcium can be obtained by drinking 2 glasses of milk/day	True	*n* = 150 (79.4%)	*n* = 49 (20.6%)
12. Moderate alcohol consumption has negative effects on the onset of osteoporosis	False	*n* = 145 (76.7%)	*n* = 44 (23.3%)
13. There are currently effective treatments for osteoporosis	True	*n* = 186 (98.4%)	*n* = 3 (1.6%)

We also evaluated the effect of some of the studied variables on the score of osteoporosis knowledge level (Table 4). Women showed a significantly higher level of knowledge about osteoporosis compared to men (*p* = 0.008). Patients with a family history of osteoporosis also scored a significant higher score compared to those without a family history, with an average score of 9.6 versus 8.8 correct responses, respectively (*p* = 0.05). The participants’ level of knowledge about osteoporosis was unaffected by age, residence, education level, recent falls, osteoporosis diagnosis, BMI and sources of information. Additionally, a positive correlation (rho = 0.153, *p* = 0.036) was observed between knowledge level score and duration of daily physical activity, suggesting that respondents that know more about osteoporosis engage in more daily physical activity.

**Table 4:** Differential analysis of osteoporosis knowledge level scores in groups of subjects.

Variable	Group	Group count (*n*=)	Mean of knowledge level	Standard deviation of knowledge level	*p*-value
Gender	Male	40	7.8	2.98	0.008^*^
	Female	149	9.2	1.87	
Age range	40–49	40	8.8	2.22	0.355
	50–59	58	9.1	1.71	
	60–69	60	8.9	2.16	
	70+	31	8.7	3.03	
Residency	Rural	58	9.2	2.24	0.259
	Urban	131	8.8	2.19	
Level of education	Secondary school	25	8.5	2.79	0.637
	High-school	76	9.1	2.17	
	Post-secondary school	22	8.7	1.86	
	University	66	8.9-	2.13	
Recent falls	No	143	8.8	2.33	0.231
	Yes	46	9.2	1.75	
Family history of osteoporosis	No	157	8.8	2.26	0.050^*^
	Yes	32	9.6	1.77	
Osteoporosis diagnosis	No	161	8.8	2.19	0.081
	Yes	28	9.6	2.20	
BMI	Underweight (<18.5 kg/m^2^)	3	10.3	0.58	0.450
	Normal weight (18.5–24.9 kg/m^2^)	51	9.0	2.40	
	Overweight (25–29.9 kg/m^2^)	84	9.0	2.23	
	Obesity (>30 kg/m^2^)	51	8.6	2.01	
Source of information	Physician	37	9.5	1.98	0.588
	Friends/family	36	9.0	1.60	
	Social networking	39	9.1	2.01	
	Others (radio/TV)	29	9.0	1.94	

^*^Statistical significance.

Next points in the survey evaluated participants’ risk of developing osteoporosis and potential prevention practices (Table 2). A significant proportion of subjects were overweight (44.5%) or obese (27.0%), with an overall average BMI of 27.6 ± 4.55. About a quarter (24.3%) of respondents have suffered a recent fall, with falls from a standing height (14.8%) being the most prevalent, being thus predisposed to fractures. Family history of osteoporosis was recorded in 16.9% of participants. In terms of osteoporosis prevention practices and lifestyle behaviors, more participants reported using vitamin D supplements (38.6%) compared to calcium supplements (28.6%). Alcohol consumption was reported by a quarter (27%) of subjects, while caffeine consumption is much more frequent, being practiced by three quarters (76.7%) of participants. Less than one-quarter (21.7%) of respondents were current smokers. When it comes to daily physical activity, most respondents (45.5%) reported doing less than 30 min of exercise per day, while only 23.8% practice more than 60 min per day.

In our study group, the majority (77.2%) of participants reported not having undergone a DXA bone quality test, the main diagnostic approach for osteoporosis (Table 2). Only 28 subjects (14.8%) undertook such an investigation once, while very small percentages made DXA test two (*n* = 7, 3.7%), three (*n* = 5, 2.6%), four (*n* = 2, 1.1%), or five (*n* = 1, 0.5%) times. Analysis of participants groups presented in Table 5 revealed that women perform this test at a higher rate than men, with statistically significant differences between the sexes (*p* = 0.03). Patients with a history of fractures performed significantly (*p* = 0.00) more DXA test (40.8%) compared to those without a history (16.4%).

**Table 5:** Differential analysis of DXA test performance in groups of subjects.

Variable	Group	DXA test = no		DXA test = yes		*p*-value
		Count (*n*=)	%	Count (*n*=)	%	
Gender	Male	36	90.0%	4	10.0%	0.030^*^
	Female	110	73.8%	39	26.2%	
Residency	Rural	45	77.6%	13	22.4%	0.941
	Urban	101	77.1%	30	22.9%	
Level of education	Secondary school	18	72.0%	7	28.0%	0.506
	High-school	56	73.7%	20	26.3%	
	Post-secondary school	19	86.4%	3	13.6%	
	University	53	80.3%	13	19.7%	
Recent falls	No	112	78.3%	31	21.7%	0.535
	Yes	34	73.9%	12	26.1%	
History of fractures	No	117	83.6%	23	16.4%	0.000^*^
	Yes	29	59.2%	20	40.8%	

^*^Statistical significance.

Out of the total number of subjects, 14.8% (*n* = 28) reported having an osteoporosis diagnosis, with the highest number of osteoporosis cases being in the 60–69 age group (57%, *n* = 16). However, 43% (*n* = 12) of individuals diagnosed with osteoporosis did not receive treatment. We then comparatively evaluated subgroups of individuals with and without osteoporosis (Table 6). There was a significant difference (*p* = 0.002) in the prevalence of the pathology across age groups, with the highest number of cases (26.7%, *n* = 16) recorded in individuals aged 60–69 (*n* = 60). The chi-square test yielded very high statistical significance for both comparisons (*p* = 0.000), indicating a strong association between osteoporosis diagnosis and the use of calcium and vitamin D supplements.

**Table 6:** Differences in preventive practices and lifestyle behaviors between diagnosed and undiagnosed respondents.

Variable	Group	Osteoporosis diagnosis = no		Osteoporosis diagnosis = yes		*p*-value (chi square test)
		Count (*n*=)	%	Count (*n*=)	%	
Age-range	40–49	40	100.0%	0	0.0%	0.030^*^
	50–59	52	89.7%	6	10.3%	
	60–69	44	73.3%	16	26.7%	
	70+	25	80.6%	6	19.4%	
Daily physical activity	<30 min/day	77	89.5%	9	10.5%	0.284
	30–60 min/day	48	82.8%	10	17.2%	
	>60 min/day	36	80.0%	9	20.0%	
Calcium supplements	No	124	91.9%	11	8.1%	0.000^*^
	Yes	37	68.5%	17	31.5%	
Vitamin D supplements	No	108	93.1%	8	6.9%	0.000^*^
	Yes	53	72.6%	20	27.4%	

^*^Statistical significance.

Lastly, we evaluated the effect of the history of certain co-associated pathologies (diabetes mellitus, hypo/hyperthyroidism, kidney problems, rheumatoid arthritis) and the use of proton pump inhibitors (PPI) on osteoporosis (Table 7). A significant higher prevalence (*p* = 0.008) of osteoporosis was recorded in patients having associated pathologies (25.5%) compared to respondents without co-morbidities (10.4%). Although osteoporosis diagnosis was more frequent in respondents taking proton pump inhibitors for more than 3 months than those who did not follow such chronic treatment (22.9% vs. 13.0%), the difference was not statistically significant. Also, no differences in the daily duration of exercise between diagnosed and undiagnosed individuals were registered. Conversely, patients suffering from associated pathologies were statistically more likely (*p* = 0.036) to have experienced falls (34.5%) compared to those without co-morbidities (20.1%).

**Table 7:** The effect of associated pathologies and the use of proton pump inhibitors on osteoporosis diagnosis and risk factors.

Variable	Group	Response = no		Response = yes		*p*-value
		Count (*n*=)	%	Count (*n*=)	%	
Osteoporosis diagnosis						
Associated pathologies	No	120	89.6%	14	10.4%	0.008^*^
	Yes	41	74.5%	14	25.5%	
PPI administration >3 months	No	134	87.0%	20	13.0%	0.138
	Yes	27	77.1%	8	22.9%	
Recent falls						
Associated pathologies	No	107	79.9%	27	20.1%	0.036^*^
	Yes	36	65.5%	19	34.5%	
PPI administration >3 months	No	117	76.0%	37	24.0%	0.834
	Yes	26	74.3%	9	25.7%	
History of fractures						
Associated pathologies	No	103	76.9%	31	23.1%	0.172
	Yes	37	67.3%	18	32.7%	
PPI administration >3 months	No	114	74.0%	40	26.0%	0.975
	Yes	26	74.3%	9	25.7%	

^*^Statistical significance; PPI, proton pump inhibitors.

Community 1 consists of 60 participants; its defining features are overweight BMI (1.17 times the overall network percentage), urban (1.41), and highly educated (2.13) participants, with no vitamin D supplements (1.86). The remaining features are irrelevant to the segregation of Community 1. We maintain the same description for the other communities. Community 2 includes 57 participants, characterized by mainly people aged 60–69 (1.58 times the average percentage) and 80–89 (2.4), males (1.34), urban (1.38) and lower educated (1.85), smokers (1.45) and coffee drinkers (1.45). Community 3, consisting of 34 participants, is characterized by participants aged between 70–79 (1.78 times the reference percentage), rural (3.35), lower educated (1.78), and engaged in physical activity more than 60 min daily (2.57), who tend to have fractures (1.29) and comorbidities (1.42), with osteoporosis (1.66), taking osteoporosis treatment (1.68), and with DEXA test (1.58). Community 4 includes 19 participants, and its most relevant features are age between 40–49 (2.96), rural (3.45), highly educated (2.13), smokers (1.23), coffee (1.23) and alcoholic beverages (1.23) drinkers, with no history of fractures (1.27) and comorbidities (1.26) and not having diagnosis of osteoporosis (1.17). Community 5 comprises 8 participants, all females (1.24 times the reference percentage), aged between 50–59 (2.76), urban (1.41) and lower educated (1.89), consuming calcium (3.52) and vitamin D supplements (2.52), engaged in more than 60 min of daily physical activity (1.56), with at least one DXA scan (1.67) and having treatment for osteoporosis (1.43). Community 6, the smallest community with five subjects, has as the main features participants aged 50–59 (3.21), underweight (24.39), urban (1.41) and with higher education (1.70), calcium (3.52) and vitamin D (2.65) supplements consumers, alcohol (2.66) and coffee (1.30) drinkers, smokers (1.30), with a history of fractures (3.18) and comorbidities (2.07), diagnostic of osteoporosis (7.04), DXA scanned (4.46) and osteoporosis treatment (6.86).

**Figure 2:**
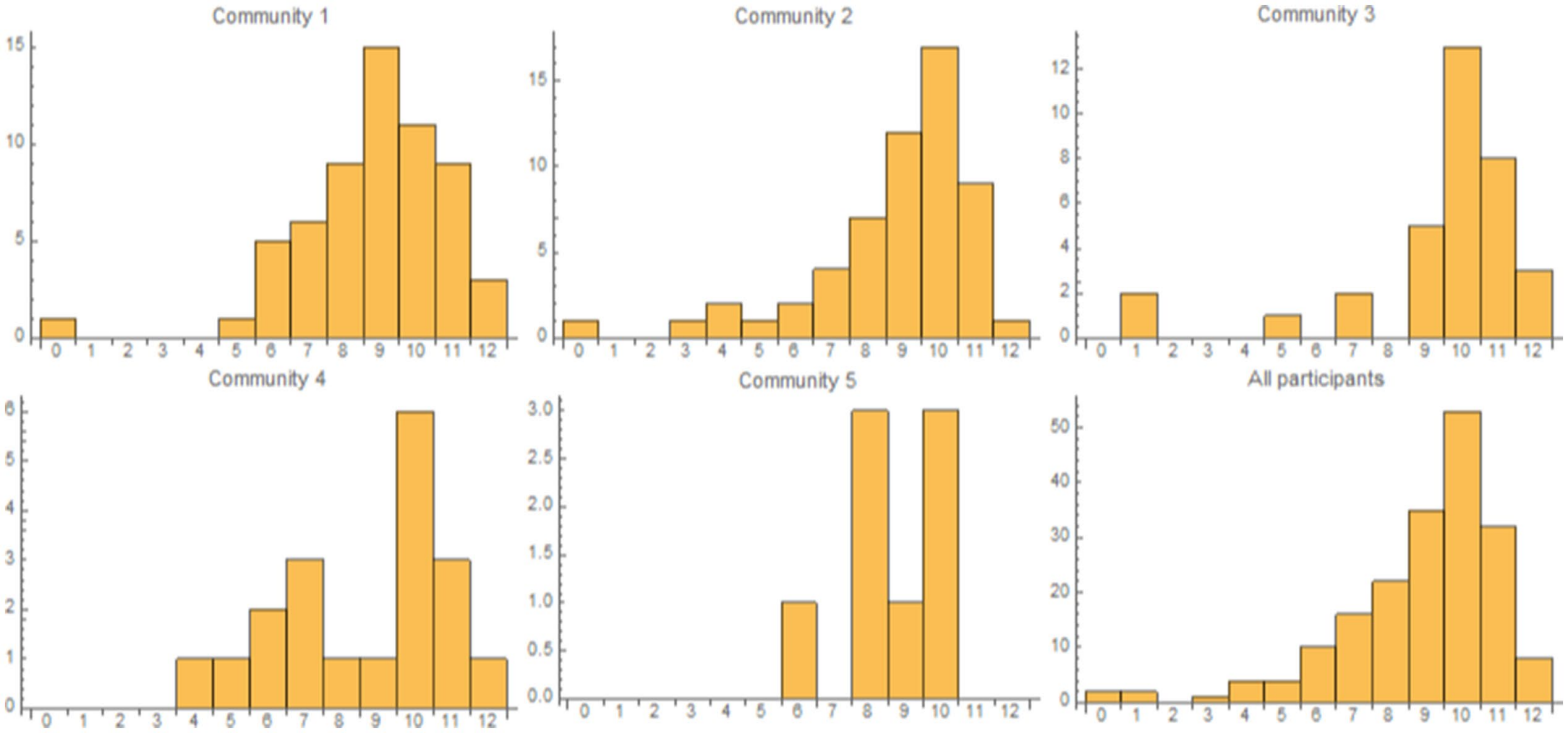
The panels present the histograms for the first 5 communities in network G and all participants, where the bins correspond to the questionnaire results values (0 to 12). We excluded Community 6 because it comprises only 5 vertices. The osteoporosis-knowledge questionnaire result distributions for each community are similar to the overall (i.e., all participants) distribution.

When investigating if there is any correlation or link between the communities from Figure 1 (described above) and the questionnaire results, we observe that the per-community distributions of results (values between 0 and 12) are similar to the overall distribution (see the histograms in Figure 2, where some of them are affected by the low number of participants). Accordingly, we cannot support any connection between the considered features—from the 4 feature classes—and the osteoporosis knowledge level revealed by the questionnaire results.

We provide a more detailed description of all communities in Supplementary Table S1.

## 4 Discussion

According to Eurostat projections, by 2060, the Romanian population over 65 years will reach 35%. Moreover, given the tendency of decreasing birth rate and the growing prevalence of CVDs, cancer, respiratory diseases, and other morbidities that require polymedication, an increase in the incidence of frailty syndrome among Romanian population is expected (31, 32). Yet, fragility research is still scarce in Romania (2). It is well-recognized that senescence plays an important role in the development of age-related osteoporosis and is directly tied to the rise in the number of frail people. Osteoporosis and CVDs are also closely linked: patients with low bone mineral density or increased bone turnover have a higher risk of frailty and therefore of cardiovascular morbidity and mortality. Thus, a multidisciplinary approach is needed in order to decrease the socio-economic burden, the iatrogenic-induced harm, the number of falls and hospitalizations in frail elderly people, and thus decrease the mortality rate (33). Our study aimed to increase clarity among healthcare providers about osteoporosis awareness in the Romanian population, with the objective of providing clear and targeted recommendations to address the identified gaps.

In terms of knowledge about osteoporosis, 75.1% of participants reported being aware of this pathology. The overall level of knowledge is moderate (9.0 ± 0.15), consistent with the findings of other studies (34–36). Compared to men, women tend to be better informed about the pathology. This gender gap has been previously documented and could be due to the misconception that osteoporosis is a women’s disease (37–41). Thus, to avoid underdiagnosis among males, it is essential to raise awareness equally in both sexes (42, 43). In contrast to the findings of previous research studies, our sample shows no statistically significant differences in the level of knowledge among respondents based on their age decade, level of education, or residency (44–48). Unexpectedly, participants with a reported diagnosis of osteoporosis did not have a better knowledge score than those without a diagnosis. This finding is consistent with a study conducted in Poland and suggests that patients could benefit from more intensive therapeutical education, possibly offered by clinical pharmacists (49). A meta-analysis published in 2020 concluded that osteoporosis preventive education could also benefit adolescents in terms of long-term bone health behaviors (50). On the other hand, respondents with a family history of osteoporosis are better informed compared to those with no family history of osteoporosis. Although primary care specialists may be more inclined to provide more information to patients with a family background of osteoporosis (51, 52), 60.8% of our respondents reported that their GP did not inform them about the risk of developing osteoporosis, a concern also identified in other studies (53–55). Instead, the main source of information regarding osteoporosis identified in our study was social networking (27.7%), followed by physician (26.2%). Thus, the underdiagnosis of osteoporosis can also be attributed to the acquisition of incomplete or misleading information about the disease from unauthorized sources (56).

The analysis of the subjects’ responses to each question assessing their level of knowledge yielded some concerning findings. Almost half of the respondents were unaware that a family history of osteoporosis increases the susceptibility to the disease, and nearly 60% considered that osteoporosis causes symptoms prior to a fracture occurring. These results imply that patients may underestimate and underinvestigate the disease, leading to a delayed diagnosis that is often established after the occurrence of potentially disabling fractures. This is further corroborated by the high percentage of respondents (77.2%) who have never undergone at least one DXA test in their lifetime. What is more, 73.9% of our respondents reported recent falls. This could relate to the fact that 64.0% of participants believed that a fall is not as important as a decrease in bone mineral density in the occurrence of a fracture. Yet again, the DXA test is performed to a significantly higher extent by women than men, supporting the assumption that men tend to be underdiagnosed. Concerningly, a recent study in Romania showed that the number of DXA scans decreased by 37.8% after the COVID-19 pandemic compared to the previous year, increasing the burden of osteoporotic fractures (57–60). Taken together, our findings suggest DXA investigations are not frequently used, possibly indicating a reduced awareness of their importance or barriers in accessing them.

There is a noticeable tendency to disregard preventive strategies in the management of osteoporosis. Calcium and vitamin D play crucial roles in bone homeostasis. Additionally, vitamin D is pivotal in calcium metabolism, facilitating its absorption (13, 61). The general recommendations for calcium intake are 1,000 mg per day for males aged 19 to 70 and females aged 19 to 50. For males aged 71 and older and for women aged 51 and older, the daily calcium intake should be 1,200 mg (13, 15, 61). International Osteoporosis Foundation recommends a vitamin D intake sufficient to maintain a serum 25(OH)D level above 20 ng/mL, with daily doses of vitamin D ranging from 800 to 1,000 IU (31). Concerningly, in Europe, there is an inadequate intake of calcium and vitamin D in the elderly, predisposing these individuals to osteoporosis (4, 61). Our results showed statistically significant differences (*p* = 0.000) for calcium and vitamin D supplementation between respondents with a diagnosis of osteoporosis and healthy respondents, with individuals already diagnosed having a greater propensity to use such supplements to reach the target level. This leads to the conclusion that calcium and vitamin D supplementation is under-utilized as a preventive measure in the Romanian population. Moreover, the majority of individuals in our study are sedentary and overweight, 44.5% of respondents having a BMI between 25 and 30 kg/m^2^. Only 23.8% of individuals reported engaging in physical activities for more than 60 min per day. A recent systematic review which included a total of 59 studies concluded that physical activity lasting more than 60 min 2–3 times/week for at least 7 months may improve bone mineral density in people 65 years old and over (14, 62). There is a positive correlation between osteoporosis knowledge and daily physical activity: respondents with higher knowledge scores tend to have a longer duration of daily physical activity (rho = 0.153, *p* = 0.036). Yet, despite the study population demonstrating moderate knowledge about osteoporosis and its risk factors, preventive measures were adopted to a limited extent, consistent with findings from other studies (40, 56, 63). Thus, the findings further underscore the necessity for both preventive and curative therapeutic education in order to enhance diagnosis rates and achieve effective management of the disease.

In our study, the overall prevalence of osteoporosis diagnosis was 14.8%. Compared with healthy subjects, respondents with associated comorbidities were more likely to experience recent falls (*p* = 0.036) or to have an osteoporosis diagnosis (*p* = 0.008), aligning with findings from previous investigations (2, 33). Alarmingly, 42.9% of respondents reported having osteoporosis did not undergo treatment, despite the fact that majority of participants stating that they were aware of effective osteoporosis treatments. In Romania, the number of individuals at high fracture risk who do not receive antiosteoporotic therapy is notably higher compared to the rest of the European countries. By 2034, a projected 15% increase in the number of fragility fractures is expected, negatively impacting the healthcare budget (15). Taking into consideration the costs per patient associated with osteoporotic fractures, Romania ranks last among the 29 European countries (15, 32).

Oral bisphosphonates (e.g., alendronate, zoledronic acid, risedronate, but not ibandronate) are recommended as the initial treatment in high-risk patients, with denosumab being considered an alternative therapy to reduce fracture risk. Teriparatide or abaloparatide for less than 2 years or romosozumab for 1 year should be the first line therapy for very high-risk patients (13, 64). Sequential therapy, involving initial treatment with a bone forming agent followed by an antiresorptive agent, are recommended to achieve better improvement in bone microarchitecture and increase BMD (13, 64). The reasons why patients at risk of fractures do not initiate antiosteoporotic treatment are numerous (1): insufficient information about the pathology and its treatment, resulting in a reduced perception of risk (2), the attitude of the general practitioner, who may lack confidence in treatment effectiveness or are concerned about potential side effects (3), reluctance towards medication, and (4) concerns regarding side effects (51, 65).

Given that osteoporosis is asymptomatic until the occurrence of the first fragility fracture (that happen even with minor trauma, such as fall from a standing position or a vertebral compression fracture incident), an efficient prevention strategy is the main key in reducing the incidence of fractures (16, 32, 66, 67). In both preventive and curative therapeutic patient education, the medical-pharmaceutical team (including physicians, nurses, pharmacists, clinical pharmacists, as well as physiotherapists, occupational therapists and dieticians) must collaborate for a more holistic approach to managing the disease. Thus, healthcare providers could achieve this goal by (1): increasing the number of informative and awareness campaigns (2), improving the identification of individuals at risk of osteoporosis (3), playing a more active role in counseling patients on supplements and promoting a healthy lifestyle (4), addressing non-adherence to medication (5), providing guidance on the proper use of anti-osteoporosis medication, and (6) reassuring patients about the benefits of the treatment and the risks associated with not taking it (68). Moreover, for patients with polypathology and consequently polypharmacy, there is a compelling need to review the therapeutic regimen to identify drugs that increase the risk of falls or may potentially induce osteoporosis over time (69–72).

The present study should be interpreted in the context of its strengths and limitations. The cross-sectional design of this study is a limitation because (1) it does not allow for the assessment of the long-term effects of osteoporosis knowledge level on osteoporosis prevention measures, and (2) osteoporosis incidence was not assessed longitudinally (3). Another limitation is the relatively small sample size, which might have negatively impacted the statistical power of certain results and (4) the limited number of patients >70 years old (aspect which will be addressed in further studies we plan to conduct on the frailty of Romanian patients). Moreover, the present findings are representative for the areas where the study was conducted and cannot be generalized for the entire country. Despite these limitations, this study is the first attempt in Romania to assess the level of knowledge among the population regarding osteoporosis and its risk factors. Furthermore, a subsidiary purpose of the present study was to raise awareness regarding the disease and its complications. The importance of the study lies in its potential to identify relevant problems and improve both the diagnosis and the treatment rates of osteoporosis.

## 5 Conclusion

Taken together, the current work reveals a moderate level of knowledge about osteoporosis in conjunction with poorly osteoprotective practices. The growing number of elderly people in Romania, coupled with the high incidence of CVDs and the presence of multiple co-morbidities such as osteoporosis collectively increase the risk of developing frailty syndrome, the primary predisposing factor to disability. In this context, it is imperative to enhance awareness and knowledge about these diseases, and to implement appropriate management strategies for polypathologies and polymedication. This will contribute to improving the quality of life of Romanian patients and consequently reduce healthcare-related costs.

## Data Availability

The raw data supporting the conclusions of this article will be made available by the authors, without undue reservation.
