# Supplementary material for: Osteoporosis: a problem still faulty addressed by the Romanian healthcare system. Results of a questionnaire survey of people aged 40 years and over
Source: Front Med (Lausanne). 2024 Oct 23;11:1485382. doi: 10.3389/fmed.2024.1485382 (PMC11537941; doi:10.3389/fmed.2024.1485382)
Supplement: Supplementary file 1 [file Table_1.pdf]

Supplementary Material

1 Supplementary Figures and Tables

Table S1. The patient features and distribution in the six communities. The first two columns present each feature's name, classification level, and the number of segregated participants. The following columns show the participants (in number and relative fraction) from each community with a particular feature.

**Feature**	**Feature's interval / level**	**Community 1 (*n* = 60)**	**Community 2 (*n* = 57)**	**Community 3 (*n* = 34)**	**Community 4 (*n* = 19)**	**Community 5 (*n* = 8)**	**Community 6 (*n* = 5)**
Age (years)	40-49 (n = 39)	17 (1.33)	7 (0.58)	3 (0.41)	12 (2.96)	-	-
	50-59 (n = 58)	18 (0.95)	15 (0.83)	15 (1.39)	3 (0.50)	7 (2.76)	
	60-69 (n = 57)	14 (0.75)	28 (1.58)	7 (0.66)	3 (0.51)	-	5 (3.21)
	70-79 (n = 25)	11 (1.39)	4 (0.53)	8 (1.78)	1 (0.40)	1 (0.94)	-
	80-89 (n = 4)	-	3 (2.40)	1 (1.34)	-	-	-
Sex	Males (n = 36)	13 (1.10)	15 (1.34)	3 (0.45)	4 (1.07)	-	1 (1.02)
	Females (n = 147)	47 (0.98)	42 (1.14)	31 (1.14)	15 (0.98)	8 (1.24)	4 (1.00)
BMI (kg/m^2)	≤18.49 (n = 3)	-	1 (1.07)	-	-	-	2 (24.39)

	18.5 – 24.99 (n = 49)	15 (0.93)	18 (1.18)	12 (1.32)	3 (0.59)	1 (0.47)	-
	25 – 29.99 (n = 81	31 (1.17)	21 (0.83)	12 (0.80)	10 (1.19)	4 (1.13)	3 (1.36)
	≥30 (n = 50)	14 (0.85)	17 (1.09)	10 (1.08)	6 (1.16)	3 (1.37)	-
Education level	Low (n = 97)	-	56 (1.85)	32 (1.78)	-	8 (1.89)	1 (0.38)
	High (n = 86)	60 (2.13)	1 (0.04)	2 (0.13)	19 (2.13)	-	4 (1.70)
Living environment	Urban (n = 130)	60 (1.41)	56 (1.38)	1 (0.04)	-	8 (1.41)	5 (1.41)
	Rural (n = 53)	-	1 (0.06)	33 (3.35)	19 (3.45)	-	-
Calcium supplements	Yes (n = 52)	19 (1.11)	6 (0.37)	12 (1.29)	2 (0.37)	8 (3.52)	5 (3.52)
	No (n = 131)	41 (0.95)	51(1.25)	22 (0.90)	17 (1.25)	-	-
Vitamin D supplements	Yes (n = 69)	24 (1.06)	13 (0.61)	15 (1.17)	5 (0.70)	7 (2.32)	5 (2.65)
	No (n = 114)	36 (1.86)	44 (1.24)	19 (0.90)	14 (1.18)	1 (0.20)	-
Alcohol consumption	Yes (n = 55)	21 (1.16)	17 (0.99)	6 (0.59)	7 (1.23)	-	4 (2.66)
	No (n = 128)	39 (0.93)	40 (1.00)	28 (1.18)	12 (0.90)	8 (1.43)	1 (0.29)

Coffee consumption	Yes (n = 141)	44 (0.95)	38 (0.87)	29 (1.11)	18 (1.23)	7 (1.14)	5 (1.30)
	No (n = 42)	16 (1.16)	19 (1.45)	5 (0.64)	1 (0.23)	1 (0.54)	-
Smoking	Yes (n = 141)	44 (0.95)	38 (0.87)	29 (1.11)	18 (1.23)	7 (1.14)	5 (1.30)
	No (n = 42)	16 (1.16)	19 (1.45)	5 (0.64)	1 (0.23)	1 (0.54)	-
Daily physical activity	≤ 60 minutes (n = 139)	53 (1.16)	49 (1.13)	13 (0.50)	15 (1.04)	5 (0.82)	4 (1.05)
	> 60 minutes (n = 44)	7 (0.49)	8 (0.58)	21 (2.57)	4 (0.88)	3 (1.56)	1 (0.83)
Fracture history	Yes (n = 46)	12 (0.80)	16 (1.11)	11 (1.29)	1 (0.21)	2 (0.99)	4 (3.18)
	No (n = 137)	48 (1.07)	41 (0.96)	23 (0.90)	18 (1.27)	6 (1.00)	1 (0.27)
Comorbidities	Yes (n = 53)	17 (0.98)	17 (1.03)	14 (1.42)	2 (0.36)	-	3 (2.07)
	No (n = 130)	43 (1.01)	40 (0.99)	20 (0.83)	17 (1.26)	8 (1.41)	2 (0.56)
Diagnosis of osteoporosis	Yes (n = 26)	4 (0.47)	8 (0.99)	8 (1.66)	-	1 (0.88)	7 (7.04)
	No (n = 157)	56 (1.09)	49 (1.00)	26 (0.89)	19 (1.17)	7 (1.02)	-
DXA	Yes (n = 41)	7 (0.52)	13 (1.02)	12 (1.58)	1 (0.23)	3 (1.67)	5 (4.46)

	No (n = 142)	53 (1.14)	44 (0.99)	22 (0.83)	18 (1.22)	5 (0.81)	-
Osteoporosis treatment	Yes (n = 16)	1 (0.19)	6 (1.20)	5 (1.68)	-	1 (1.43)	3 (6.86)
	No (n = 167)	59 (1.08)	51 (0.98)	29 (0.93)	19 (1.10)	7 (0.96)	2 (0.44)
